# Toxic Metals Depuration Profiles from a Population Adjacent to a Military Target Range (Vieques) and Main Island Puerto Rico

**DOI:** 10.3390/ijerph17010264

**Published:** 2019-12-30

**Authors:** Héctor Jirau-Colón, Ashley Cosme, Víctor Marcial-Vega, Braulio Jiménez-Vélez

**Affiliations:** 1 Department of Biochemistry, School of Medicine, Medical Sciences Campus, University of Puerto Rico, San Juan 00936, Puerto Rico; hector.jirau@upr.edu; 2 Center for Environmental and Toxicological Research, San Juan 00936, Puerto Rico; Ashley.cosme1@upr.edu (A.C.); marcialvegamd@aol.com (V.M.-V.); 3 Department of Radiology, School of Medicine, Universal Central del Caribe, Bayamón 00956, Puerto Rico

**Keywords:** heavy metals, metals, environmental toxicology, military activity, pollution, environmental pollution

## Abstract

**Background**: The island of Vieques (a municipality of Puerto Rico) was used as a military practice range by the US Navy for more than 60 years. Many studies have reported the presence of toxic metals in soil samples taken from Vieques. The bombing range is only 18 km upwind from the Vieques residential area and inhalable resuspended particles resulting from bombing are known to reach the populated area. The current study reports for the first time, the presence of toxic metals’ depuration profiles obtained from Vieques and Main Island Puerto Rico human subjects. **Objectives**: This study was designed to evaluate the distribution of toxic metals in a random population exposed to contaminants originating from military activities and comparing it to a non-exposed random population from Main Island Puerto Rico. **Methods**: A total of 83 subjects studied; 32 were from Vieques and 51 were from Main Island Puerto Rico. A physician administrated chelation therapy to all subjects and collected urine samples during a 24-h period. A total of 20 trace elements associated with military activities were measured in urine by induced coupled plasma mass spectrometry (ICP-MS). The results were compared between both population samples. **Results**: Significant differences in the levels of eight trace elements associated with military practices were found between Vieques and Main Island Puerto Rico. Lead (Pb), aluminum (Al), uranium (U) (*p* < 0.001), arsenic (As), cadmium (Cd) (*p* = 0.02), and gadolinium (Gd) (*p* = 0.03) were significantly higher in Vieques while niobium (Nb) and platinum (Pt) levels (*p* < 0.006) were lower in the Vieques samples. **Discussion**: Higher concentrations of Pb, Al, As, Cd, Gd, and U were found in Vieques residents’ urine samples compared to Main Island. Nonetheless, Pt and Ga were present in Main Island at higher concentrations than in Vieques. Although limited by its sample size, this report should set a basis for the importance of health assessment in these subjects exposed to military activities remnants throughout the years and further evaluation of their effects on the overall health of the population.

## 1. Introduction

**Figure 1:**
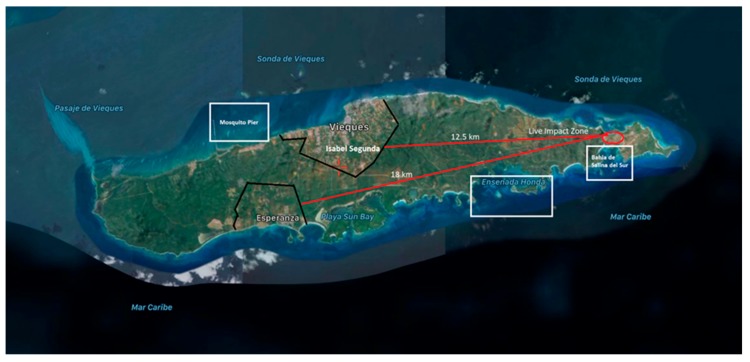
The island of Vieques used as a military bombing range. The live impact zone is illustrated with a red circle right above Bahia Salinas del Sur. Two big communities are located on the island, in the north (Isabel Segunda), which is about 12.5 km from the impact range (red line), and in the south (Esperanza), about 18 km from the live impact zone. The three white rectangles represent fishing areas where queen conch was collected for toxic metal analysis.

**Figure 2:**
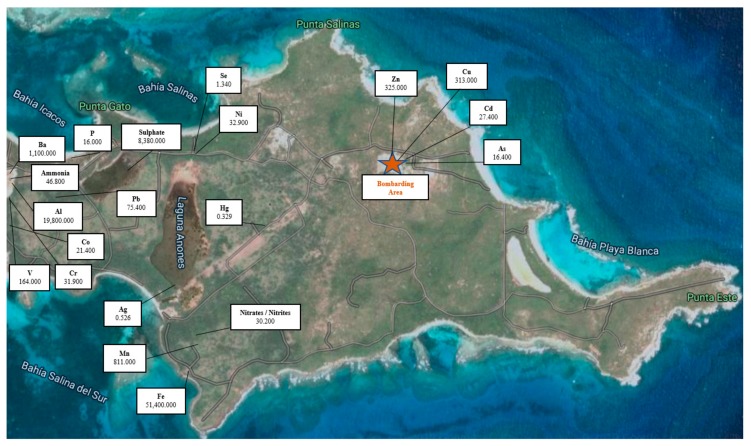
Toxic metal concentrations in soil within the impact bombing range on the east coast of Vieques Island. The concentrations of each metal are shown in the white rectangles (values are shown in parts per million). Source: Neftalí Garcia et al., 2000; samples taken by Scientific and Technical Services (SCT, Inc.); Digital image, USGS, 1994.

Towards the end of the 1930s, the US Navy began to acquire land on the Puerto Rican municipality of Vieques for the purpose of establishing a military bombing training area. These military facilities were near two civilian populations (which are 13 to 18 km from the bombing range) (Figure 1). The east side of Vieques island became a live ammunition target range used until 2003 (60 years of military activity), and consequently, the adjacent soil, air, and marine environment became contaminated with toxic metal concentrations [1,2,3] (Figure 2). Frequent military exercises around the world in areas in proximity to civilian populations are known to generate environmental pollution [4]. The east of Vieques was devoted to ammunition military practices, including napalm, agent orange, and depleted uranium ammo [5]. Explosive bombs, on the Eastside, were continuously dropped for 60 years only 13 to 18 km west from the island inhabitant population. After the bomb detonations, the resuspended particles can reach a height of 100 m [6]. Assuming an average diameter of 5 µm (respirable particles), the average settling velocity of a fallout particle, with a density of 2.7 g cm^−3^, as calculated using Stokes law and assuming a dynamic shape factor of 1.3, would be about 0.16 cm s^−1^ [7]. Therefore, on average, the time required for the redeposition of resuspended fallout particles (respirable size) from the bombing cloud at a height of 100 m would be about 17 h. The average wind velocity in Vieques is 16 km/h and the direction is from east to west (https://weatherspark.com/y/28057/Average-Weather-in-Vieques-Puerto-Rico-Year-Round). This is more than enough time for the resuspended particles to reach the adjacent population, thus allowing exposure to dust originating from explosions [8].

For years, the nearby Vieques population was breathing particles that remained airborne during the detonation due to military practices (reaching the populated area a little after an hour). Therefore, it is evident that Vieques residents were exposed during the 60 years (military activity) to polluted ambient resuspended particles originating from military bombing. Although the Center for Disease Control and Prevention (CDC), the United States health protection agency, public health assessment reports [9] that the resuspended particle material does not pose a health risk to the population, we believe that the concentration of contaminants in ambient particulate matter (PM) should have been determined and used for the health risk assessment. However, the PM data, including airborne metal concentration, are inexistent for Vieques. Even though many of these pollutants are known carcinogens, the residents of Vieques have not been monitored for the body burden of environmental chemicals to this date [5,10]. Nevertheless, a later sampling study (on metals) was performed on 500 residents in 2004 and reported by the Puerto Rico Health Department [11].

Previous studies performed on the residential crop and other soft commodities in Vieques assessed the presence of toxic metals. The results showed significantly higher levels of metals in Vieques as compared to the Main Island Puerto Rico [12]. Furthermore, high levels of toxic metals have been reported in soil samples taken by the United States Geological Survey (USGS) (1994) at the impact bombing range (see Figure 2) [13]. The data gathered in this study are consistent with the hypothesis that toxic metals originating at the target range are airborne and transported to civilian areas (Figure 1) by windblown dust resuspended at the bombing zone.

Toxic metals, such as depleted uranium, lead, and gadolinium, are prevalent in the environment due to their use as a kinetic energy penetrator, modern bullet-design, and use in alloys of iron and chromium to improve resistance to high temperatures and to prevent oxidation of military equipment [14]. Many of these metals have no biological function and have been reported to interfere with biochemical pathways [15,16]. Studies by Massol-Deya compared different trace metals in the seagrass *Syringodium filiforme* collected at the bombing ranges with those similar under geo-climatic conditions. The results revealed higher bioaccumulation of toxic metals near the military bombing site. Lead (Pb) and cadmium (Cd) concentrations in seagrass *Syringodium filiforme* showed a bioaccumulation effect in the US Navy military site when compared to non-military activity sites [17]. Therefore, due to the proximity of the military impact range to the human population and evident exposure to toxic metals, it is of utmost importance to evaluate and compare the relative abundance of specific toxic components with a sub-population from the Main Island of Puerto Rico. The transfer of toxic constituents through common environmental sources (food, air, water supply, etc.) are possible routes of health concern. We have found no other studies done in adjacent populations to similar military bombing ranges. The only comparable site we are aware of is on the island of Kaho’olawe, which housed a military bombing range during World War II through the 1990s when the site was transferred to the jurisdiction of the state of Hawaii. Furthermore, after ending its military use as a bombing range, many environmental issues remain, and the health risk uncertainty is at question.

The *American Journal of Public Health* reported that the cancer rates on Vieques were low (from 1960 to 1979) when compared to the Main Island of Puerto Rico, yet the cancer rates for the period 1985–1994 increased [10]. Figueroa et al. (2009) found that tumor distribution changes between Vieques and Main Island Puerto Rico were greatest in prostate, colorectal cancer, and, particularly, in lung and bronchial cancer, with 17% in Vieques and only 8% in the Main Island population. For the year 2000, observed cancer cases were 26% higher than expected using the Main Island as a reference [18]. The present study was designed to test for the presence of toxic metals’ depuration profiles related to military activity in the Vieques population compared to a reference site in Puerto Rico mainland. This was achieved by comparing urine toxic metal depuration profiles between a subset of the Vieques population and a similar population from the Main Island of Puerto Rico.

## 2. Materials and Methods

### 2.1. Study Design and Sample Selection

This study was designed using voluntary human subjects’ members of two clinics, one in the municipality of Vieques and the other in the Main Island of Puerto Rico. Main Island subjects have not been exposed to the military remnants found in Vieques nor other facilities. Our goal was to collect 24-h urine samples after a chelating treatment and compare urine toxic metal concentrations in these two individual populations. The cohort was nested in a population-based randomized trial from patients from Vieques and from the San Juan area of Puerto Rico during 2014. Sampling sites were established as follows: The geographical location in Vieques corresponds to a village near the military site where subject recruitment was performed; our control population were subjects from the Municipality of San Juan randomly recruited from a voluntary clinic. A total of 83 urine samples were collected (51 from the Main Island and 32 from Vieques). Recruitment was difficult since subjects participating in the chelating therapy were on a voluntary basis and hence, limited. Since participation in this study was strictly based on voluntary individual availability and providing consent to participate in the metal-chelating therapy, the population size was limited.

The Vieques population sample consisted of 37% females from the following four residential regions: Isabel II (43.2%), Florida (21.4%), Esperanza (21.4%), and Puerto Ferro (14%). The Main Island population (Puerto Rico) consists of a pool of 51 subjects, 43% female and resident in the capital, San Juan. The urine collection and shipping protocol was conducted according to the Genova Comprehensive Urine Element Profile & Toxic Element Clearance Profile (Genova Diagnostics, Ashville, NC, USA, see website cited in the references section). There were no children considered in the study, thus all individuals were 21 years of age or older.

### 2.2. EDTA Ca^2+^ Chelating Therapy and Urine Collection

The study consisted of administering subjects an Intravenous (IV) chelating therapy using calcium Ethylenediaminetetraacetic acid (EDTA), followed by a 24-h urine collection period, and a subsequent induced coupled plasma mass spectrometry (ICP-MS) toxic metal analysis to determine metal concentrations. Neither subject information nor personal identifier was available to anyone other than the lead physician to keep participants and samples hidden from any person performing the data analysis (blinded); thus, our study was based solely on the sample number identifier, gender, age, and site information.

The depuration procedure consisted of a 5 to 10-min IV push injection of undiluted calcium EDTA 300 mg/cc and 5 to 10 cc of ozone 20 μg/cc of ozone oxygen mixture. The therapy was administered through a 23-gauge butterfly infusion needle, and the calcium EDTA into the vein without any dilution [19]. The calcium EDTA dose used was 50 mg per kg of body weight. This procedure was performed by a trained community registered nurse at a local community center or the physician in Vieques and in Main Island Puerto Rico. After the administered therapy, the Genova Diagnostics’ Comprehensive Urine Elements Profile and Toxic Element Clearance Profile urine kits were used to collect urine specimens [20]. Subjects were instructed to collect their urine during a 24 h period, starting with the first urine immediately after injection up until the first urine of the following day. During this time, the samples were stored in a 3-L plastic container refrigerated at 4 °C. After the 24-h collection period, the total volume of the sample was recorded, and the container was inverted and vortexed to homogenize the solution as directed in the urine kit’s protocol. The urine homogenate was transferred into two (15 mL) conical centrifuge tubes. Specimens were identified with an identifier number and sent to Genova Diagnostics Laboratories Inc. (Ashville, NC, USA) for ICP-MS trace metal profiling.

### 2.3. Toxic Metal Analysis

Genova Diagnostics Inc. (Ashville, NC, USA) analyzed all urine samples using their standard validated ICP-MS protocol (www.gdx.net). The assay for elemental analysis has been developed and its performance characteristics have been well established and validated by the company. Elemental reference ranges were developed from a healthy population under non-provoked/non-challenged conditions. A total of 20 elements in the urine sample cohorts were determined (rubidium (Rb), aluminum (Al), arsenic (As), barium (Ba), mercury (Hg), nickel (Ni), tin (Sn), cadmium (Cd), lead (Pb), antimony (Sb), thallium (Tl), tungsten (W), gallium (Ga), platinum (Pt), uranium (U), bismuth (Bi), cesium (Ce), gadolinium (Gd), niobium (Nb), and thorium (Th) (Table 1). These elements were chosen due to their use in most military practices. The element concentration was obtained in μg/g of creatinine and subsequently converted to μg/L of urine (using the mean creatine value in blood).

### 2.4. Statistical Analysis

Statistical analyses were performed using the software Minitab 17 Statistical Software (Minitab, Inc, State College, PA, USA.) and GraphPad Prism version 8.0.0 for Windows (GraphPad Software, San Diego, CA, USA). Data distribution was evaluated using box plots with Tukey whiskers. To assess potential variations in the concentration of elements in urine, we used Friedman’s test. Toxic metals with significant differences (CI: 95%, *p* < 0.05) between samples were further assessed using the Mann–Whitney test to evaluate potential differences due to outliers. The Mann–Whitney U test was used to include ranges and median comparations between age groups and gender given the unmatched sample size. The Chi-square test was used to determine outliers, thus sample values within Q < 0.01 were considered outliers and not included in our assessment of the median elemental concentrations for each population, nor used to assess statistical significance between samples.

## 3. Results

The toxic metals’ median concentrations and ranges (in parenthesis) in all urine samples (*n* = 83) are shown in Table 1. The largest differences in the following elements were found in urine samples from Vieques: Lead, aluminum, uranium (*p* < 0.001), arsenic, cadmium (*p* = 0.02), and gallium (*p* = 0.003) when compared with those of the Main Island. However, platinum (*p* < 0.006) was lower in Vieques compared to Main Island and niobium was not found in urine at all. No significant differences were observed in other toxic metals between locations. The levels determined for Ce, Ni, and Rb were slightly higher in the Main Island, but these differences were not statistically significant. Lead (Pb) values in the Vieques samples were almost five-fold higher than in those of Main Island (5.70 vs. 1.05 μg/L), with Vieques subjects exhibiting urine levels as high as 20 μg/L. Conversely, the median values of platinum were slightly higher in Main Island (0.012 versus 0.000 μg/L). A similar pattern was detected for gadolinium (0.07 vs. 0.02 μg/L (Table 1). Thorium (Th) was not found in any of the 83 depuration profiles nor were any notable differences in Hg, Sb, Ba, Ce, Ni, Tl, Sn, W, and Rb urine content noted between locations. Interestingly, Bi was detected in two Vieques samples at considerably high levels (41.65 and 2.44 μg/L). After analyzing for outliers (Q < 0.1), only the 2.44 μg/L value was considered; thus, we are not able to compare urine concentrations of bismuth between populations accurately.

**Figure 3:**
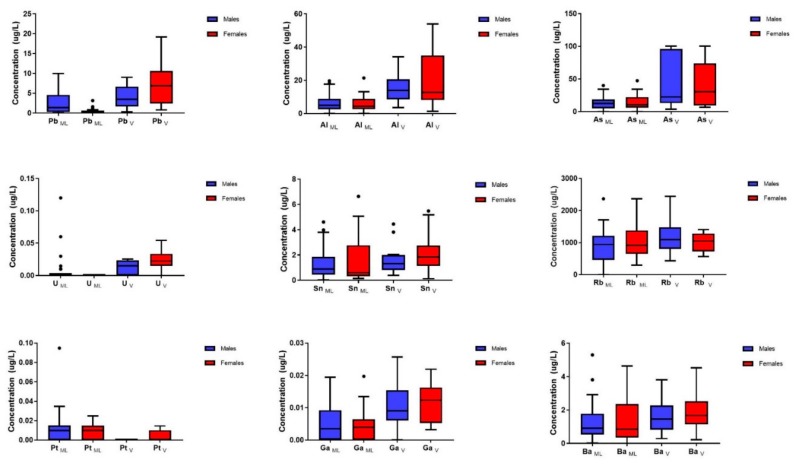
Box plots for toxic metal concentrations in the urine of subjects after chelation therapy, using gender comparison between Main Island and Vieques population. The boxes represent the first and third quartiles (inner quartile range; IQR), and the inside line represents the median. The whiskers represent the lowest and highest value within 1.5 times the IQR. Dots represent outliers.

Eight Vieques urines’ toxic metal depuration profiles (Pb, Al, As, Cd, Gd, Ga, Pt, and U) were significantly different from those from Main Island Puerto Rico (Table 1). Uranium was found in significantly higher concentrations in samples from Vieques compared to samples from the Main Island. Further analyses revealed gender differences in the urine metal content between locations (Figure 3). The highest concentrations of Pb, Al, and U were found in female urine samples from Vieques (levels as high as 20, 50, and 0.05 μg/L, respectively) when compared to females in the Main Island. Platinum concentrations in subjects from the Main Island were notably higher than those in Vieques; nevertheless, females from Vieques had higher levels than males. Urine levels of both Ga and Ba in all Vieques subjects were relatively higher than in the Main Island. In summary, many urine toxic metals were found to be higher in Vieques female subjects compared to their male counterparts.

To further evaluate the urine depuration profiles data, we examined the potential differences between age and sites. We separated all samples into two main age groups (34–54 and 55–75) (Table 2). Initial analysis revealed that irrespective of age, six elements (Pb, Al, As, Ga, Sn, and U) were significantly higher in Vieques depuration profiles when compared to those of Main Island, Puerto Rico. Conversely, platinum was found to be higher in the Main Island (0.01 μg/L with a level as high as 47.92 μg/L) compared to Vieques (0.00 μg/L and a maximum of 0.05 μg/L). This difference in depuration profiles can be attributed to the older age group, 55–75. High levels of Cd, between Vieques and Main Island (0.59 μg/L vs. 0.14 μg/L), Tl (0.15 μg/L vs. 0.08 μg/L), and Sn (1.33 μg/L vs. 0.45 μg/L), respectively, were seen; when the analysis considered age, these were strongly associated with the 34–54 age group. Uranium was found in median concentrations of 0.03 μg/L in Vieques for the 34–54 age group and at 0.02 μg/L for the 55–75 age group. The following trace elements, Pb, Hg, Al, As, Cd, Sn, Rb, and U, were higher in the Vieques age group of 55 to 75 when compared to Main Island Puerto Rico.

Additionally, we performed a correlation analysis between all metals analyzed at each of the two locations. We considered a cut off in a coefficient of correlation (R) above 0.70 to be a substantial strong correlation. Any correlation lower than 0.70 was not considered to be strong. A total of four relatively strong correlations between trace elements were found in Vieques. Rb and Cs had the highest correlation with an R of 0.83; these elements have very similar properties in nature. Pt and Ni had an R of 0.76, Cd and Al (R of 0.73), and finally, Tl and Cs with an R of 0.70. A total of five relatively strong correlations between trace elements in mainland Puerto Rico were encountered. The strongest correlation was found between Cd and Pb with an R of 0.88, making it the strongest correlation between metals found at either of the two sites. There is another strong correlation between Pb and Al in the Main Island with an R of 0.78, and U and Pb also correlate with an R of 0.70. Cd and Al also correlated very well with an R of 0.77. Rb and Cs are also correlated in the Main Island, as also seen in Vieques with an R of 0.72. Overall, there were only two strong metal correlations that are shared between both sites: Cd/Al and Rb/Cs.

## 4. Discussion

Although many of the toxic metals examined in this project are normally found in the environment, these can be greatly enriched in environments impacted by military activities. For over 60 years, as much as 200 days of bombing per year took place on the island of Vieques, where residents are settled downwind from the old military target range. Virtually every conventional and non-conventional weapon was used in Vieques by the United States Navy from 1940 until 2003. This includes napalm, agent orange, depleted uranium (1999), white phosphorous, chemical weapons, and tons of high explosives and minute particles of a fiber-glass type substance, known as “chaff” [21]. Vieques does not have a main natural source of water since it does not have a main river and the precipitation on the island is approximately 42 inches per year. Therefore, in 1977, an aqueduct was built to pump water from the mainland (Naguabo) to the island. In addition to water provided by the PR water authority, Vieques also consumes groundwater obtained from private and public sources. A study conducted by ATSDR in 2013 states that water from public sources does not present a health problem. ATSDR also states that the air pollution model employed indicates that they originate from the military site activities [22]. Several studies have previously reported high levels of toxic metals in the area of the impacted target range in Vieques. Among these metals, we found Zinc and Cu [13]. In another comprehensive study published by A. Massol and E. Diaz (2000) on Vieques, high levels of toxic metals in various environmental compartments, including the impacted area, and high Pb and Cd levels were reported [23].

When studying the metal depuration profiles obtained from subjects from Vieques and the reference site in Puerto Rico mainland, we found two strong metal correlations (above R 0.70) that are shared between sites. One of these correlations is between Rb and Cs and the other between Cd and Al. The Rb and Cs correlation is stronger for Vieques residents (R of 0.83 vs. R of 0.72 for the Main Island). Rb and Cs are elements that are usually found together because they behave similarly in nature with similar properties [24]. Therefore, with these similar characteristics, they are expected to be closely related in both environments. The other strong metal relationship found at both sites is between Cd and Al. These two metals are found related in cigarette smoke and therefore this could be a variable associated with these two metals [25]. However, the levels of Al and Cd in Vieques samples are much higher than those from the Main Island and are associated with females and in the age group of 34–54 years, suggesting that there is an additional variable involved with the Vieques residents. Aluminum and lead and their sources are discussed further in the section below. Pt and Ni found in Vieques residents’ urine are also correlated, but it is important to note that Pt concentrations in urine are very low, but Ni is high in the age group of 34 to 54 in Vieques. Although these metal correlations of Tl/Cs, and Pt/Ni are only found in urine from Vieques residents, the reasons for these correlations are unknown.

The most common diseases in Vieques as reported by ATSDR 2013 are hypertension, asthma, diabetes, allergies, arthritis, and heart diseases. An unusually high lung and bronchial cancer incidence in the Vieques municipality has been reported [5,22]. The ATSDR 2013 study also reports a lung cancer incidence ratio of 2.25 between the years of 1990 and 1995 [22].

The presence of arsenic has been associated with cancer [26,27] and it has been detected above the US Environmental Protection Agency (EPA) standards in the oil in areas close to the military bombing site in Vieques. As much as 16.400 ppm of arsenic was reported in the Vieques bombing area (Figure 2) and as high as 20 ppm [2], and it is also elevated in vegetation at the Vieques bombarding area, [13,28]. An epidemiological study was performed by the Puerto Rican Health Department in 2004, which is the most extensive human biomonitoring study conducted to date in Vieques [11]. The study tested 500 Vieques residents and analyzed the levels of arsenic, cadmium, and nickel in hair and urine; Al, Pb, and Hg in blood; and uranium in urine. The reference value for As in urine is less than 50 µg/L [29]. In this study, the geometric mean for the total As in the urine of 500 residents was 33.6 µg/L. In total, 23% of the population tested were above 50 µg/L. Thirty-nine residents who did not consume fish had levels above 50 µg/L. It is evident that As exposure is transferred through fish consumption [30]. A pilot study conducted in 2006, evaluated whether greater seafood consumption from Vieques-Puerto Rico is associated with increased exposure to As [31]. Nail, hair, and urine samples were used as biomarkers of inorganic As exposure in adult women and men. Only the concentration of As in nails was a good biomarker for As transfer through fish consumption. It is also noticed that urinary excretion of As in the Vieques population was higher than that found in other countries. The study concludes that fish consumption does not contribute significantly to As burden in the Vieques population. Therefore, the As burden found in the Vieques population must be attributed to other sources, such as airborne and other food consumption. Our depuration study shows that males had higher concentrations of arsenic than Vieques females and greater than in either gender in the Main Island. This suggests that the body burden for As and the bioaccumulation in the system is much higher in the Vieques population compared to the rest of the world. Therefore, it provides a reason to suspect that military activities performed on the island are linked to the reason for such exposure.

Levels of mercury in the soil in the impact area have been reported at the highest level of 0.086 ppm in soil [32]. Normal blood Hg values are considered as 10 µg/L [33]. A 2009 survey reports 1 µg/L as the average blood concentration for Hg in the US population [29]. The maximum level of Hg detected in the blood from 500 Vieques residents was 16 µg/L [11]. The presence of mercury in female hair samples was also reported as high [34]. Trace elements gradually bioaccumulate and persist in different organ tissues as they are gradually excreted (depending on the individual excretion rates). Very few studies, however, have evaluated the impact of military activities on Vieques and have considered the bioavailability of trace elements to residential communities downwind and adjacent to the military site(s). A study performed in Vieques describes high levels of toxic metals of Hg in hair and nails from local individuals [34]. Levels of Hg could be attributed to various sources, including fish food, and hence, it recommends not eating fish during pregnancy. Adding to these studies, we evaluated urine toxic metals eliminated through depuration trials from two distinct populations, one close to the military range in Vieques and the other from the Main Island of Puerto Rico. Our studies conclusively show that toxic metals, such as Pb, Al, As, Cd, Ga, Pt, and U, associated with military activities were found to be in significantly higher concentrations in Vieques residents compared to those from Main Island Puerto Rico. However, we did not find significant differences in Hg depuration levels between Vieques and PR mainland [34,35].

Lead (Pb) is the most common element in ammunition and used during military practice [1,12]. Data provided by the EPA Discharge Monitoring Reports by the Atlantic Fleet Weapons Training Facility (AFWTF) of inorganic compounds show high levels of lead and arsenic in waters surrounding the impacted area [2]. Levels on the order of 75.4 (Figure 2) and 33 ppm have been reported in the soil at the impact site [32]. High levels of lead were detected in Vieques vegetation when compared to Main Island Puerto Rico, particularly in pasture grass where levels were above safety guidelines [28]. The 95th percentile in blood Pb concentration for Vieques children (1 and older) from 1999 to 2004 was 4.9 µg/dL [29]. The recommended Pb blood standard for children is 5 µg/dL [36,37]. We found that the Pb concentrations are five-fold higher in Vieques urine depuration samples when compared to the Main Island (Table 1). The levels of lead found in the population through the Vieques urine depuration profiles could be attributed as a result of an enhancement of re-suspended and downwind transported particulate matter that is either inhaled or deposited in residential soils and vegetation, increasing human and livestock exposure to toxic metals. In addition, the transfer of toxic metals from contaminated sediments to the marine environment and bioaccumulation in aquatic organisms are also critical routes (primary economy of Vieques) for the transfer of these pollutants (such as As and Hg).

Aluminum is known to be a principal constituent of military equipment, including chaffs (radar countermeasure). The average normal level of Al in the blood is 1 to 3 µg/L [38]. In this study, the geometric mean for blood aluminum was reported at 17.6 μg/L. Aluminum blood levels over 400 subjects were found to have values above 10 µg/L. Most of Vieques residents had over twice these levels. In total, 22% (109 persons) showed Al blood levels above 40 µg/L and 10 persons had levels greater than 60 µg/L. Most of the residents with levels higher than 40 were in the ages between 20 and 44. We found that the Al content was approximately two-fold higher in the depuration profile of Vieques (14.20 µg/L) compared to the Main Island (7.95 µg/L). However, the greatest depuration of Al was associated with a higher age group of 55 to 75 years.

The U.S. Navy confirmed the use of depleted uranium (U) in these military bombarding sites [39]. The normal levels of uranium in urine are 0.5 μg/L. The PRDOH 2006 study only identifies uranium in only six of the 500 resident samples [11]. The geometric mean of these six participants was 0.14 μg U/L in urine. All six participants were women. The depuration profiles obtained for Vieques participants also show the highest levels of U associated with females. A notable difference between median concentrations of uranium in Vieques depuration profiles (higher) was found when compared to Main Island (a few outliers present, but no consistency among them) (see Figure 3). The possibility that these differences between U in the two populations are associated with food consumption (additives) is likely since the diet of Vieques’ residents is much likely to be higher in seafood (due to local availability) compared to that of the Main Island. Seafood consumed by the residents in Vieques originates from local fishing while the sources of seafood for residents of Main Island come from various locations. It is important to consider that uranium accumulates in aquatic organisms, hence the significance of fishing as a possible source for toxic metal transfer across the food chain [40]. The bioaccumulation of uranium in marine organisms is generally low. Algae and plankton can accumulate U at concentrations in the order of 1 to 10 µg/g d.w. [41,42] while mollusks and shellfish can accumulate U at a range of 0.0020 to 0.3 μg/g d.w. [42,43]. A NOAA report made public in 2016 revealed the levels of uranium in the marine queen conch *Strombus Gigas* collected at various locations within Vieques [8]. Out of a total of 21 queen conches, 13 of them close to the impact site and 3 in the far northwest (see Figure 1) had a concentration ranging from 0.13 to 0.83 µg/g of wet weight (mean of 0.491 µg/g). The highest U concentrations were found in conches closest to the target range, suggesting site proximity-related differences in exposure. Similar results were obtained for Cd levels in tissues of queen conch by sites.

Further analyses of our data compared the presence of toxic metals by gender from Vieques and Main Island. As previously mentioned, Pb, Al, As, U, and Ga concentrations were higher in the Vieques population, with gender-related differences in metal urine contents. Females from Vieques had higher concentrations of most trace elements when compared to males from either Vieques or the Main Island. Lead, aluminum, and uranium were significantly higher in females from Vieques compared to both Vieques males and Main Island (Table 1). It has been documented that toxic metals, such as Ni, Pb, Hg, and As, are elevated in soil and vegetation at the Vieques bombarding area [13,28]. Hg has also been found in women of reproductive age from Vieques [34]. In the present study, platinum was unexpectedly found to be in significantly higher concentrations in the urine of Main Island subjects when compared to Vieques residents (see Table 1).

In addition, the toxic metal depuration metabolic profiles of two age groups (34–54 and 55–75) (Table 2) exhibited significant differences between populations. Lead, aluminum, arsenic, gallium, tin, and uranium were significantly higher in Vieques than in Main Island, irrespective of the age group. However, these metals were elevated in females compared to males from the same site. The 34–54 age group exhibited higher concentrations for 50% of the toxic metals tested while the 55–75 age group showed 41% of toxic metals with a notable difference in depuration profiles. Thallium was found to be significantly higher in the younger age category (34–54) while no significant difference was found in the higher age group in Vieques nor Main Island.

## 5. Conclusions

The present study reports the presence of various toxic elements in human subjects exposed on an island that was used as a military target from 1947 to 2003 compared to an unexposed population. Higher concentrations of lead, aluminum, arsenic, cadmium, gallium, and uranium were found in Vieques residents’ urine samples compared to Main Island. Aluminum, Pb, and As have been reported in relatively high concentrations in the residents of Vieques [11]. Nonetheless, platinum and gadolinium were present in Main Island at higher concentrations than in Vieques. Correlations between elements in their respective sampling populations were analyzed but not discussed given the small sample size and the possibility of a nonlinear relationship among the variables. Nonetheless, this was included as Supplementary Materials as we intend to further explore these relationships and expand the coverage of our project. There are various limitations to this study, mostly revolving around the sample size. Since this process was done on voluntary subjects that came to the clinics, it was difficult to enroll more patients to match the population with the Main Island, which had a higher sample size due to better accessibility and a higher overall population living there. Nevertheless, this report should set a basis for the importance of health assessment in these subjects exposed to military activities throughout the years and further evaluation of its effects on the overall health of the population. We look forward to continuing expanding our work on Vieques and further determine associations among elements, source of exposure, and a more in-depth assessment of the health effects of exposure to these toxic metals.

## Figures and Tables

**Table 1:** Median element concentrations (range) in urine samples from patients in Main Island (*n* = 51) and Vieques (*n* = 32), Puerto Rico.

Element	Mainland (μg/L)	Vieques (μg/L)	*p*-Value
Aluminiun	7.95 (0.00–30.10)	14.20 (1.80–67.80)	<0.001
Antimony	0.038 (0.00–0.18)	0.05 (0.00–1.33)	0.456
Arsenic	16.00 (0.00–82.00)	22.50 (7.00–175.00)	0.016
Barium	1.50 (0.00–6.70)	1.50 (0.30–13.80)	0.376
Bismuth	0.00 (0.00–6.21)	0.00 (0.00–54.85)	0.892
Cadmium	0.24 (0.00–5.06)	0.43 (0.09–1.55)	0.016
Cesium	5.10 (0.22–16.55)	4.30 (2.20–12.80)	0.165
Gadolinium	0.07 (0.00–20.85)	0.02 (0.00–2.45)	0.028
Gallium	0.006 (0.00–0.037)	0.01 (0.00–0.03)	0.003
Lead	1.050 (0.10–13.60)	5.70 (0.40–37.60)	<0.001
Mercury	0.60 (0.00–7.43)	0.71 (0.00–1.90)	0.648
Nickel	0.85 (0.00–15.39)	0.42 (0.00–17.16)	0.083
Niobium	0.00 (0.00–0.37)	0.00 (0.00)	0.006
Platinum	0.01 (0.00–3.34)	0.00 (0.00–0.13)	0.006
Rubidium	(0.20–3488.00)	1136.50 (546.00–2393.00)	0.354
Thalium	0.15 (0.01–0.88)	0.14 (0.07–0.37)	0.153
Thorium	0.00 (0.00)	0.00 (0.00)	NA ^1^
Tin	1.18 (0.09–12.11)	1.67 (0.16–123.08)	0.090
Tungsten	0.00 (0.00–0.20)	0.05 (0.00–0.32)	0.812
Uranium	0.00 (0.00–0.20)	0.02 (0.00–0.05)	0.001

^1^ NA = Not available.

**Table 2:** Median toxic metal concentrations (range) distributed by age groups 34–54 and 55–74 in Main Island and Vieques.

	Age Group 34–54 (Years Old)			Age Group 55–75 (Years Old)		
	Mainland (ug/L)	Vieques (ug/L)	*p*-Value	Mainland (ug/L)	Vieques (ug/L)	*p*-Value
Elements						
Lead	0.29 (0.023–6.28)	6.60 (0.46–19.16)	0.000	1.46 (0.10–16.55)	7.93 (0.83–46.05)	0.003
Mercury	0.39 (0.00–1.75)	0.814 (0.00–1.99)	0.560	0.38 (0.00–2.61)	0.73 (0.00–2.33)	0.099
Aluminum	4.16 (0.18–32.83)	10.86 (1.33–53.94)	0.004	6.29 (0.00–18.25)	16.13 (3.78–81.37)	0.000
Antimony	0.04 (0.00–0.09)	0.05 (0.02–0.10)	0.013	0.03 (0.00–0.18)	0.04 (0.00–0.08)	0.379
Arsenic	10.78 (0.23–165.75)	27.20 (6.86–100.15)	0.017	14.79 (0.00–99.59)	59.40 (3.90–100.30)	0.007
Barium	0.85 (0.02–5.18)	1.95 (0.23–10.42)	0.101	0.82 (0.00–5.30)	1.46 (0.67–6.37)	0.081
Bismuth	0.00 (0.00–0.82)	0.00 (0.00–41.65)	0.606	0.00 (0.00–3.53)	0.00 (0.00–0.00)	
Cadmium	0.14 (0.00–0.43)	0.59 (0.07–1.80)	0.000	0.22 (0.03–1.43)	0.54 (0.13–2.45)	0.120
Cesium	2.41 (0.06–6.33)	4.53 (1.90–15.85)	0.101	3.40 (1.06–7.89)	4.64 (2.41–6.13)	0.114
Gadolinium	0.01 (0.00–100.00)	0.03 (0.00–0.09)	0.754	0.04 (0.00–91.81)	0.02 (0.00–3.14)	0.108
Gallium	0.01 (0.00–0.01)	0.01 (0.00–0.03)	0.001	0.00 (0.00–0.02)	0.01 (0.00–0.03)	0.001
Nickel	0.00 (0.00–1.77)	0.49 (0.00–2.24)	0.450	0.60 (0.00–5.20)	0.17 (0.00–11.64)	0.174
Niobium	0.00 (0.00)	0.00 (0.00)		0.00 (0.00–0.05)	0.00 (0.00)	
Platinum	0.01 (0.00–0.08)	0.00 (0.00–0.05)	0.146	0.01 (0.00–47.92)	0.00 (0.00–0.09)	0.027
Rubidium	817 (15–1479)	1052 (461–4361)	0.136	940.20 (0.10–1776.10)	1070 (437–2443)	0.072
Thalium	0.08 (0.00–0.21)	0.15 (0.06–0.42)	0.028	0.12 (0.00–0.23)	0.13 (0.04–0.35)	0.316
Thorium	0.00 (0.00)	0.00 (0.00)		0.00 (0.00)	0.00 (0.00)	
Tin	0.45 (0.02–21.16)	1.33 (0.38–93.00)	0.007	1.05 (0.17–7.29)	2.15 (0.72–47.00)	0.056
Tungsten	0.04 (0.00–0.14)	0.05 (0.00–0.24)	0.246	0.04 (0.00–0.14)	0.05 (0.00–018)	0.174
Uranium	0.00 (0.00–0.04)	0.03 (0.00–0.06)	0.001	0.00 (0.00–0.03)	0.02 (0.00–0.03)	0.019

## Supplementary Materials

The following are available online at https://www.mdpi.com/1660-4601/17/1/264/s1.
