# Supplementary material for: Toxic Metals Depuration Profiles from a Population Adjacent to a Military Target Range (Vieques) and Main Island Puerto Rico
Source: Int J Environ Res Public Health. 2019 Dec 30;17(1):264. doi: 10.3390/ijerph17010264 (PMC6994965; doi:10.3390/ijerph17010264)
Supplement: Supplementary file 1 [file ijerph-17-00264-s001.pdf]

**Table S1.** Correlations between trace elements found in urine collected from Main Island Puerto Rico subjects. Outliers were considered within Q < 0.01 and not taken into consideration in the analysis to account for extreme values.

| | **Pb** | **Hg** | **Al** | **Sb** | **As** | **Ba** | **Bi** | **Cd** | **Cs** | **Gd** | **Ga** | **Ni** | **Nb** | **Pt** | **Rb** | **Tl** | **Th** | **Sn** | **W** | **U** |
|---|---|---|---|---|---|---|---|---|---|---|---|---|---|---|---|---|---|---|---|---|
| **Pb** | **100%** | 31% | 78% | 34% | 5% | 13% | 33% | 88% | 46% | 29% | 52% | -7% | 28% | -7% | 29% | 25% | Null | 18% | 37% | 70% |
| **Hg** | 31% | **100%** | 23% | 32% | 21% | 19% | 17% | 33% | 26% | 19% | 14% | 4% | -9% | -13% | 24% | 26% | Null | -4% | 26% | 12% |
| **Al** | 78% | 23% | **100%** | 37% | 4% | 19% | 26% | 77% | 52% | 32% | 57% | 5% | 13% | -8% | 35% | 30% | Null | 7% | 37% | 67% |
| **Sb** | 34% | 32% | 37% | **100%** | 3% | 20% | 5% | 28% | 42% | 11% | 53% | 29% | -6% | -12% | 48% | 13% | Null | 25% | 56% | 27% |
| **As** | 5% | 21% | 4% | 3% | **100%** | 37% | 0% | -2% | 26% | 4% | -10% | -2% | -13% | -8% | 29% | 31% | Null | 0% | -4% | -4% |
| **Ba** | 13% | 19% | 19% | 20% | 37% | **100%** | 20% | 8% | 27% | 11% | 20% | 6% | -13% | -12% | 33% | 21% | Null | 19% | 8% | 14% |
| **Bi** | 33% | 17% | 26% | 5% | 0% | 20% | **100%** | 45% | 16% | 29% | 11% | 18% | -7% | -4% | -7% | -2% | Null | 15% | 12% | 25% |
| **Cd** | 88% | 33% | 77% | 28% | -2% | 8% | 45% | **100%** | 51% | 36% | 45% | -8% | 4% | -4% | 19% | 10% | Null | 7% | 27% | 68% |
| **Cs** | 46% | 26% | 52% | 42% | 26% | 27% | 16% | 51% | **100%** | 21% | 45% | -3% | -19% | -11% | 72% | 46% | Null | 31% | 42% | 18% |
| **Gd** | 29% | 19% | 32% | 11% | 4% | 11% | 29% | 36% | 21% | **100%** | 11% | -14% | -8% | -4% | 10% | 15% | Null | 11% | 7% | 26% |
| **Ga** | 52% | 14% | 57% | 53% | -10% | 20% | 11% | 45% | 45% | 11% | **100%** | 10% | 12% | -3% | 44% | 27% | Null | 26% | 48% | 38% |
| **Ni** | -7% | 4% | 5% | 29% | -2% | 6% | 18% | -8% | -3% | -14% | 10% | **100%** | 11% | -1% | -1% | -8% | Null | 12% | 0% | 30% |
| **Nb** | 28% | -9% | 13% | -6% | -13% | -13% | -7% | 4% | -19% | -8% | 12% | 11% | **100%** | -2% | -12% | 18% | Null | 0% | 18% | 43% |
| **Pt** | -7% | -13% | -8% | -12% | -8% | -12% | -4% | -4% | -11% | -4% | -3% | -1% | -2% | **100%** | -11% | 13% | Null | -7% | -14% | -2% |
| **Rb** | 29% | 24% | 35% | 48% | 29% | 33% | -7% | 19% | 72% | 10% | 44% | -1% | -12% | -11% | **100%** | 55% | Null | 22% | 55% | 5% |
| **Tl** | 25% | 26% | 30% | 13% | 31% | 21% | -2% | 10% | 46% | 15% | 27% | -8% | 18% | 13% | 55% | **100%** | Null | 3% | 16% | 4% |
| **Th** | Null | Null | Null | Null | Null | Null | Null | Null | Null | Null | Null | Null | Null | Null | Null | Null | Null | Null | Null | Null |
| **Sn** | 18% | -4% | 7% | 25% | 0% | 19% | 15% | 7% | 31% | 11% | 26% | 12% | 0% | -7% | 22% | 3% | Null | **100%** | 36% | 2% |
| **W** | 37% | 26% | 37% | 56% | -4% | 8% | 12% | 27% | 42% | 7% | 48% | 0% | 18% | -14% | 55% | 16% | Null | 36% | **100%** | 14% |
| **U** | 70% | 12% | 67% | 27% | -4% | 14% | 25% | 68% | 18% | 26% | 38% | 30% | 43% | -2% | 5% | 4% | Null | 2% | 14% | **100%** |

**Table S2.** Correlations between trace elements found in urine collected from Vieques subjects. Outliers were considered within Q < 0.01 and not taken into consideration in the analysis to account for extreme values.

| | **Pb** | **Hg** | **Al** | **Sb** | **As** | **Ba** | **Bi** | **Cd** | **Cs** | **Gd** | **Ga** | **Ni** | **Nb** | **Pt** | **Rb** | **Tl** | **Th** | **Sn** | **W** | **U** |
|---|---|---|---|---|---|---|---|---|---|---|---|---|---|---|---|---|---|---|---|---|
| **Pb** | **100%** | 56% | 6% | -10% | 2% | 37% | -15% | 18% | -4% | 7% | 2% | -8% | Null | 4% | -2% | 3% | Null | 0% | 3% | 27% |
| **Hg** | 56% | **100%** | 36% | -17% | 19% | 38% | -24% | 26% | 16% | 12% | 15% | -10% | Null | -10% | 13% | 24% | Null | 3% | 13% | 54% |
| **Al** | 6% | 36% | **100%** | -10% | 29% | -4% | -10% | 73% | 36% | 7% | 40% | 0% | Null | -18% | 48% | 26% | Null | 0% | 6% | 36% |
| **Sb** | -10% | -17% | -10% | **100%** | -19% | -1% | -5% | -12% | 21% | -2% | -20% | -7% | Null | -9% | 2% | -7% | Null | -6% | -9% | 4% |
| **As** | 2% | 19% | 29% | -19% | **100%** | -10% | -17% | 40% | -1% | 29% | 9% | 30% | Null | 7% | 16% | 24% | Null | -9% | -22% | -2% |
| **Ba** | 37% | 38% | -4% | -1% | -10% | **100%** | -17% | 6% | 29% | -9% | 28% | -19% | Null | -4% | 1% | 27% | Null | 63% | 19% | 7% |
| **Bi** | -15% | -24% | -10% | -5% | -17% | -17% | **100%** | -18% | -6% | -4% | 16% | -4% | Null | -5% | -5% | -15% | Null | -5% | 52% | -23% |
| **Cd** | 18% | 26% | 73% | -12% | 40% | 6% | -18% | **100%** | 42% | 19% | 44% | 22% | Null | 9% | 58% | 40% | Null | 11% | -14% | 53% |
| **Cs** | -4% | 16% | 36% | 21% | -1% | 29% | -6% | 42% | **100%** | 9% | 51% | 3% | Null | -23% | 83% | 71% | Null | 36% | 21% | 25% |
| **Gd** | 7% | 12% | 7% | -2% | 29% | -9% | -4% | 19% | 9% | **100%** | 16% | -6% | Null | -7% | 4% | 15% | Null | 0% | -8% | 14% |
| **Ga** | 2% | 15% | 40% | -20% | 9% | 28% | 16% | 44% | 51% | 16% | **100%** | 0% | Null | -4% | 36% | 37% | Null | 58% | 23% | 25% |
| **Ni** | -8% | -10% | 0% | -7% | 30% | -19% | -4% | 22% | 3% | -6% | 0% | **100%** | Null | 76% | 5% | -2% | Null | -5% | 3% | 1% |
| **Nb** | Null | Null | Null | Null | Null | Null | Null | Null | Null | Null | Null | Null | Null | Null | Null | Null | Null | Null | Null | Null |
| **Pt** | 4% | -10% | -18% | -9% | 7% | -4% | -5% | 9% | -23% | -7% | -4% | 76% | Null | **100%** | -23% | -25% | Null | -13% | -11% | 5% |
| **Rb** | -2% | 13% | 48% | 2% | 16% | 1% | -5% | 58% | 83% | 4% | 36% | 5% | Null | -23% | **100%** | 69% | Null | -2% | 4% | 27% |
| **Tl** | 3% | 24% | 26% | -7% | 24% | 27% | -15% | 40% | 71% | 15% | 37% | -2% | Null | -25% | 69% | **100%** | Null | 25% | 3% | 32% |
| **Th** | Null | Null | Null | Null | Null | Null | Null | Null | Null | Null | Null | Null | Null | Null | Null | Null | Null | Null | Null | Null |
| **Sn** | 0% | 3% | 0% | -6% | -9% | 63% | -5% | 11% | 36% | 0% | 58% | -5% | Null | -13% | -2% | 25% | Null | **100%** | 24% | -7% |
| **W** | 3% | 13% | 6% | -9% | -22% | 19% | 52% | -14% | 21% | -8% | 23% | 3% | Null | -11% | 4% | 3% | Null | 24% | **100%** | -9% |
| **U** | 27% | 54% | 36% | 4% | -2% | 7% | -23% | 53% | 25% | 14% | 25% | 1% | Null | 5% | 27% | 32% | Null | -7% | -9% | **100%** |
